# Imaging hamster model of bile duct cancer in vivo using fluorescent l-glucose derivatives

**DOI:** 10.1007/s13577-015-0131-5

**Published:** 2016-02-03

**Authors:** Hiroshi Yokoyama, Ayako Sasaki, Tadashi Yoshizawa, Hiroshi Kijima, Kenichi Hakamada, Katsuya Yamada

**Affiliations:** Department of Gastroenterological Surgery, Hirosaki University Graduate School of Medicine, Hirosaki, Aomori 036-8562 Japan; Department of Physiology, Hirosaki University Graduate School of Medicine, 5 Zaifu-cho, Hirosaki, Aomori 036-8562 Japan; Department of Pathology and Bioscience, Hirosaki University Graduate School of Medicine, Hirosaki, Aomori 036-8562 Japan

**Keywords:** l-glucose, Tumor, Imaging, Endoscope, Carcinoma

## Abstract

Extrahepatic bile duct cancer (cholangiocarcinoma) has a poor prognosis. Since surgical resection is the only way to prolong the patient’s life, it is of critical importance to correctly determine the extent of lesions. However, conventional pre-operative assessments have insufficient spatial resolution for determining the surgical margin. A fluorescent contrast agent might provide a more precise measure to identify anomalies in biliary surface, when combined with probe-based confocal laser endomicroscopy (pCLE). We have previously shown that 2-[*N*-(7-nitrobenz-2-oxa-1,3-diazol-4-yl)amino]-2-deoxy-l-glucose (2-NBDLG), a fluorescent derivative of l-glucose (fLG), is specifically taken up into spheroids consisting of cells showing heterogeneous nuclear-cytoplasm ratio, a feature of malignant cells in clinical settings. In addition, a combined use of 2-TRLG, a membrane-impermeable fLG, with 2-NBDLG visualized membrane integrity as well. We therefore explored in the present study the availability of the fLGs in vivo as a contrast agent for pCLE by using a hamster model of cholangiocarcinoma. Extrahepatic cholangiocarcinoma developed in mid common duct in ~20 % of the animals subjected to cholecystoduodenostomy with the ligation at the distal end of the common duct followed by injection of a carcinogen *N*-nitrosobis(2-oxopropyl)amine. After infusing bile duct with a solution containing 2-NBDLG and 2-TRLG, the lumen was surgically exposed and examined by pCLE. Fluorescence pattern characterized by bright spots and dark clumps was detected in the areas diagnosed with cholangiocarcinoma in later histopathology, whereas no such pattern was detected in control animals. These findings may form a basis for elucidating a potential availability of fLGs in imaging cholangiocarcinoma by pCLE.

**Electronic supplementary material:**

The online version of this article (doi:10.1007/s13577-015-0131-5) contains supplementary material, which is available to authorized users.

## Introduction

For extrahepatic cholangiocarcinoma, the only potential curative treatment currently is complete resection [1]. However, pre-operative assessments of cholangiocarcinoma tend to be associated with low sensitivity. Indeed, thin-section spiral computed tomography, magnetic resonance cholangiopancreatography (MRCP), and positron emission tomography with 2-[^18^F]fluoro-2-deoxy-d-glucose (FDG-PET) provide only limited information on surgical margin since the anomaly often spreads superficially along with the longitudinal axis of the bile duct [2]. Endoscopic retrograde cholangiopancreatography (ERCP) using conventional endoscope does not greatly improve situation even with brush cytology/forceps biopsy.

As such, surgical margin is determined practically based on the intraoperative rapid histopathologic diagnosis of the dissected tissue, which is processed in frozen sections, although such sections provide far less information compared to that produced by later paraffin-embedded sections.

Recent progress in optical imaging represented by probe-based confocal laser endomicroscopy (pCLE) might well provide more detailed information on surface anomalies [3–7]. One of key issues to be discussed when applying pCLE technique to diagnosis of cholangiocarcinoma is fluorescent contrast agents to be used [7–9]. Indeed, since fluorescein, which is widely used in most pCLE studies, does not enter into cells, precise identification of biliary lesions with fluorescein remains somewhat obscure [7, 10].

Numbers of trials have been made to improve such situation. Of these, a green fluorescence-emitting, d-glucose derivative 2-NBDG [11] is a unique tracer, which is taken up into living mammalian cells through glucose transporters (GLUTs) in a time, concentration and temperature-dependent manner [12, 13]. 2-NBDG has been successfully applied to a wide variety of cells such as pancreatic cells [12, 14], brain cells [15–18], as well as tumor cells both in vitro and in vivo [19–21]. Ex vivo studies using human biopsy specimens have further shown that 2-NBDG is effective for imaging oral, breast, and Barrett’s-associated neoplasia, where the tissue samples were incubated with 2-NBDG post excision to allow the uptake of the agent [22–24]. When applying such derivatives of naturally occurring d-glucose to cancer diagnosis in vivo, some issues still remain to be solved; how to avoid their potential toxicity to normal cells, and how to discriminate fluorescence due to uptake by non-cancerous cells and that originated from cancerous/precancerous cells [10, 24].

A fluorescent derivative of l-glucose (fLG) 2-[*N*-(7-nitrobenz-2-oxa-1,3-diazol-4-yl)amino]-2-deoxy-l-glucose (2-NBDLG) was originally developed as a negative control substrate for monitoring the stereoselectivity of glucose uptake [25]. Surprisingly, a portion of mouse insulinoma (MIN6) [26] cells took up abundant 2-NBDLG, when they formed three-dimensional spheroids consisting of cells with various nuclear-cytoplasm ratio (N/C), a major cytological criterion for cells suspected of high grade of malignancy in clinical settings [27].

However, in vivo availability of the fLG for cancer detection has yet to be fully investigated. Using derivatives of GLUT-unrecognizable l-glucose as functional probes for pCLE might well reduce background uptake into normal cells while minimizing the potential toxicity. In addition, a simultaneous use of 2-TRLG, Texas Red-bearing, membrane-impermeable fLG [28], with 2-NBDLG may help assess specific uptake and that by loss of membrane integrity often associated with cancerous lesions [27].

A hamster model of bile duct cancer has been well established by Tajima and colleagues [29]. Thus, we explored in the present study the way to apply the fLG to bile duct of the hamster and to image cholangiocarcinoma by using the fLG as a contrast agent for pCLE.

## Materials and methods

### Animals

A total of 37 (body weight, 136.4 ± 20.3 g) female Syrian golden hamsters (SLC, Shizuoka, Japan) were used in the present study. The animals were housed singly and allowed to feed and drink ad libitum. All animal studies were performed in accordance with and approved by the Animal Care and Use Committee of Hirosaki University Graduate School of Medicine.

### Reagents

Krebs–Ringer buffer (KRB) solution containing 2-NBDLG and 2-TRLG (2-NBDLG: 2-TRLG = 5:1, the fLG mixture) was prepared as reported previously [27]. In brief, 2-NBDLG (23003-v, Peptide Institute Inc. Osaka, Japan) was dissolved at a final concentration of 100 μM in KRB solution containing NaCl 129 mM, KCl 4.75 mM, KH_2_PO_4_ 1.2 mM, MgSO_4_ 1.2 mM, CaCl_2_ 1.0 mM, NaHCO_3_ 5.0 mM, d-glucose 5.6 mM, HEPES 10 mM (pH 7.35) just before experiments. A membrane-impermeable fLG bearing Texas Red (2-TRLG) was added for monitoring membrane integrity at a final concentration of 20 μM to the 2-NBDLG solution. 2-NBDLG and 2-TRLG were provided by Peptide Institute Inc. Carbenoxolone (100 μM, C4790, Sigma) was routinely added to block hemichannel/gap junction.

### Induction of bile duct tumors

Abdominal surgery was conducted for 27 animals at 7 weeks old under sodium pentobarbital anesthesia (i.p., 60 mg/kg body weight) according to the method reported by Tajima and colleagues [29]. Briefly, animals were subjected to cholecystoduodenostomy (surgical anastomosis of the gallbladder and the duodenum) with the ligation of the extrahepatic bile duct in the distal end of the common duct (CDDB) (see also Fig. 2a). Four weeks later, a carcinogen *N*-nitrosobis(2-oxopropyl)amine (BOP, Toronto Research Chemicals Inc., North York, ON, Canada) was subcutaneously administered weekly at a dose of 10 mg/kg body weight for 9 weeks to induce bile duct cancer (CDDB/BOP procedure).

### Anesthesia for the imaging

For fLG imaging, an original general anesthesia method was conducted to hamsters in the experimental group at 17-23 weeks after the beginning of BOP administration. Since hamsters particularly those subjected to CDDB/BOP procedure were extremely vulnerable to general anesthesia possibly due to hepatic lesions induced by CDDB/BOP procedure [29, 30], we used urethane (i.p. 680 mg/kg body weight) in combination with sodium pentobarbital. Practically, after the urethane treatment, a small quantity of sodium pentobarbital was repeatedly injected intraperitoneally so as not to depress respiration until a stable anesthesia was obtained (final concentration, 36 mg/kg body weight). This anesthesia procedure was completed within an hour.

Throughout the operation and imaging, the rectal temperature was kept at 36 ± 1 °C with the assistance of a heating plate (DC-MP10DM, Kitazato, Japan) and a heating lamp. During the period of infusing solution (see main text), the animal was covered by a box equipped with an infrared heating film on the ceiling to ensure dark environment and to prevent lowering of the body temperature. Successful infusion of the fLG mixture was confirmed by illuminating the suction line with a UV lamp.

### A probe-based confocal laser endomicroscopy (pCLE)

Imaging with pCLE (Cellvizio system, Mauna Kea Technologies, Paris) was successfully conducted for totally 27 animals. No difference in the body weight was detected between animals subjected to CDDB/BOP procedure (mean = 136.2 ± 19.5 g, *n* = 17, at 19–23 weeks after the beginning of BOP administration except for one at 17 weeks) and that of control animals (mean = 136.3 ± 18.3 g, *n* = 10, either 16–23 weeks old or 49–61 weeks old). Of 17 animals in CDDB/BOP group, 3 animals were used for assessing autofluorescence of the biliary epithelia in a condition with no fLG administration.

The pCLE probe used had a lateral resolution of 3.5 μm (moving single tumor cells could be resolved), a confocal depth of 0–50 μm, a field of view of 600 μm in diameter, 2.4 mm in the outer diameter at the probe end, and 1 m in length (Demo probe, Mauna Kea). In some experiments, images were taken in addition by a clinical probe (Alveoflex, Mauna Kea) as well, which has optical properties identical to those of Demo probe except for a thinner outer diameter (1.4 mm) and longer length (3 m). The calibration process of the probe automatically adjusted both the dynamic range and the absolute intensity so that highest contrast was obtained. As such, only qualitative evaluation of fluorescence pattern was possible in the present study.

### Macro zoom microscopy

During image acquisition, the position of pCLE probe attached to the bile duct surface was monitored by a CCD camera (2048 × 2048 pixels, Retiga 4000R, QImaging, BC, Canada) mounted on a modified multi-purpose, upright macro zoom fluorescence microscope (AZ100, Nikon, Tokyo) equipped with a xyz motorized stage (Prior Scientific, MA). A commercially available software (NIS-Elements, Nikon) was used for image acquisition. The lens used was CFI SFluor × 4 (NA 0.20, working distance 15.5 mm). Excitation, emission, and cutoff wavelength used for detecting 2-NBDLG (green) fluorescence were 470/40, 545/55, and 500 nm, respectively. Similarly, 567/15, 641/75, and 593 nm, were used for detecting 2-TRLG (red) fluorescence, respectively. The color lookup tables were adjusted to fixed values, 128 and 2048 for 2-NBDLG and 2-TRLG, respectively, to evaluate membrane damage adequately.

### Histology

After imaging, the bile duct was surgically removed, fixed with 20 % neutral-buffered formaldehyde, and cut into 3–6 pieces at right angles to the longitudinal axis with intervals of approximately 2 mm. The fixed pieces were sectioned at every 4 μm, subjected to the standard hematoxylin and eosin (H&E) staining, and were diagnosed by two expert pathologists for bile duct cancer. In one case in CDDB/BOP, and 7 cases in control group, the bile duct was cut into 4-µm-thick sections in parallel with the luminal surface.

### Statistics

The Freeman-Hamilton extension of Fisher’s exact test was conducted for analyzing the relationship between heterogeneity in fLG fluorescence pattern and histopathological diagnosis of fLG-administered animals in CDDB/BOP group (*n* = 14). The diagnosis was categorized into three groups; carcinoma including invasive one and carcinoma in situ (carcinoma), hyperplasia including reactive one and mild dysplasia (hyperplasia), and dysplasia for all remaining cases (dysplasia). The heterogeneity in the fLG fluorescence was classified into marked, moderate, and mild (or homogeneous) by two independent raters. Values are expressed as mean ± S.D.

## Results

### Extrahepatic cholangiocarcinoma observed in hamsters of CDDB/BOP group

Of 27 animals subjected to CDDB/BOP procedure, 14 animals were successfully imaged, histologically processed, and analyzed as the experimental group. Remaining 13 animals were used for preliminary experiments to establish methods of anesthesia, histology, and imaging including assessment of autofluorescence. 10 animals, which had no CDDB/BOP procedure, were used as a control group.

**Fig. 1:**
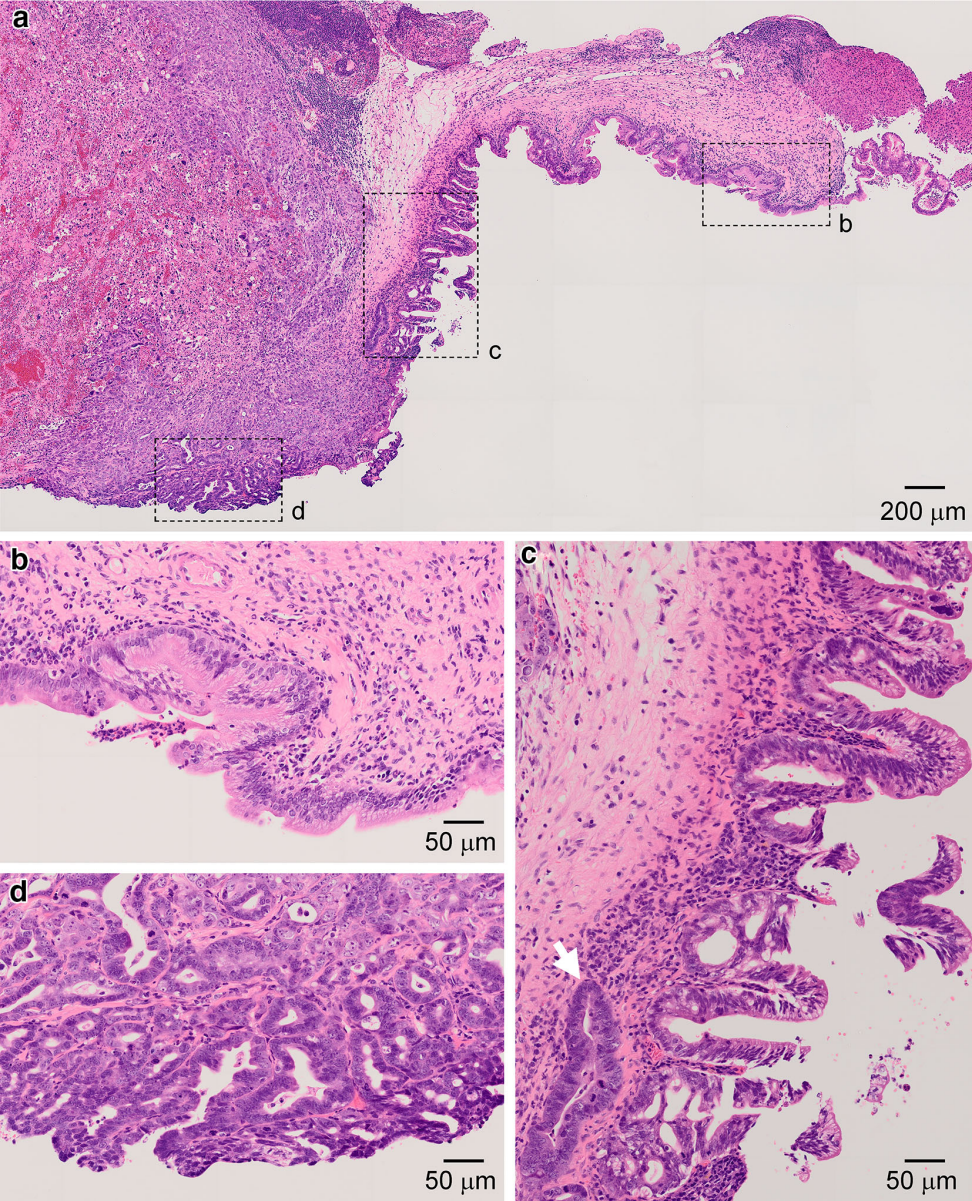
Histopathological features of bile duct cancer established in a hamster. **a** A hematoxylin and eosin (H&E)-stained cross section of the mid bile duct of a 39-week-old hamster, which was subjected to cholecystoduodenostomy with the ligation of the extrahepatic bile duct in the distal end of the common duct (CDDB) followed by administration of *N*-nitrosobis(2-oxopropyl)amine (BOP) procedure. The animal was sacrificed, fixed, and processed 29 weeks after the beginning of BOP administration.* Demarcated areas* are shown below in *magnified views*. **b** Area *b* indicated in (**a**) in a* magnified view*, showing relatively simple epithelium with some indication of hyperplasia developed in the areas adjacent to the area *b*. **c** Area *c* indicated in (**a**), showing dysplasia with highly disorganized papillary cytoarchitecture to carcinoma in situ (*lower part*). Anomalies characterized by such as loss of polarity and heterogeneously enlarged nuclei are seen (*arrow*). **d** Area *d* indicated in (**a**), showing invasive adenocarcinoma

**Fig. 2:**
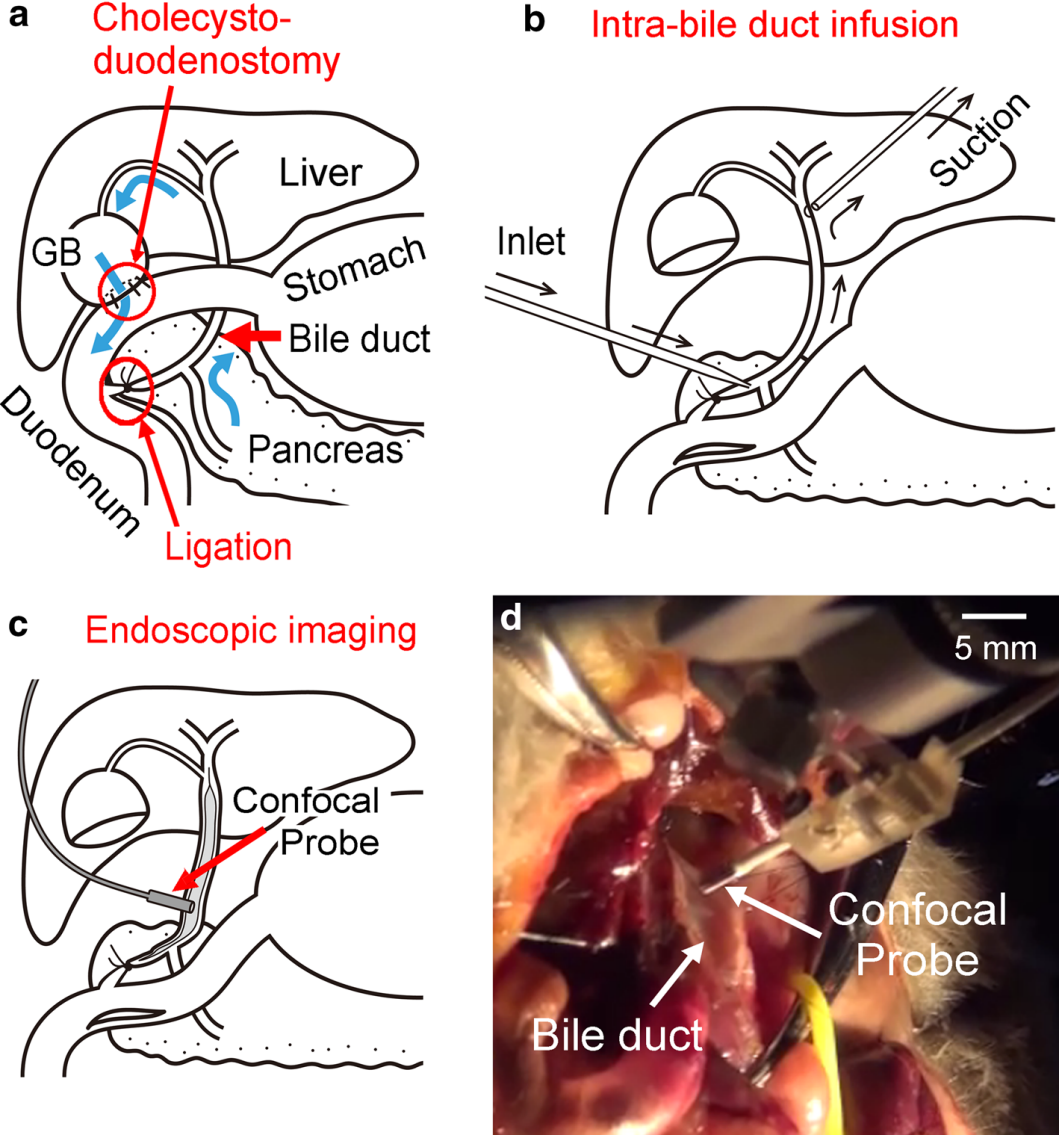
Schematic illustration of CDDB, infusion of the fLG mixture, and imaging with a probe-based confocal laser endomicroscopy (pCLE). **a** CDDB procedure. **b** Intra-bile duct infusion of the fLG. **c** Imaging excised bile duct of hamster with pCLE. **d** A photograph of the exposed bile duct being approached by the pCLE probe

In preliminary series of experiments, two out of 13 animals subjected to CDDB/BOP procedure developed invasive extrahepatic cholangiocarcinoma in mid common duct (Fig. 1). This was caused by repeated BOP administration after CDDB procedure, which produced regurgitation of pancreatic juice upward the biliary tract and its flowing into the duodenum through the gallbladder (Fig. 2a, light blue arrows. See “Materials and methods”).

Interestingly, epithelium showing severe invasive carcinoma was restricted to a particular surface of the bile duct (Fig. 1a). The cytoarchitecture gradually changed from simple epithelia, hyperplasia, dysplasia, carcinoma in situ, to invasive carcinoma in this order (Fig. 1b–d) along with the luminal surface of the bile duct. No apparent infiltration was detected in the bile duct of other 11 animals in CDDB/BOP group except two, which were diagnosed with atypia of suspicious for malignancy.

### Intra-bile duct infusion with fLG mixture

The site of cholecystoduodenostomy was surgically separated to expose the extrahepatic bile duct (Fig. 2a, b). For infusing the bile duct, blunt ended 30 G needle was carefully inserted into the lumen close to the ligation site at the distal end of the bile duct (Fig. 2b). A small incision was then made at the proximal end of the duct to aspirate the infused solution with a suction pipette connected to vacuum lines (Fig. 2b).

After initial flushing out of bile duct contents with warm KRB for 5 min, the fLG mixture was infused for 5 min at a rate of 0.3 ml/min with the assistance of a syringe pump (KDS100, KD Scientific, MA). Then, the fLG mixture remained in the lumen was washed out with KRB with no added fLG for 5 min.

### Imaging biliary surface by pCLE in vivo

After washout of the fLG mixture, lumen of the bile duct was exposed by a careful incision made longitudinally throughout the duct (Fig. 2c) followed by pulling the wall of the duct carefully in a crosswise direction with suture threads (7-0 nylon). The animal was then moved to the motorized xyz stage of an upright macro zoom fluorescence microscope while being warmed on the heating plate, then the luminal surface of the bile duct was quickly inspected (Online Resource 1).

**Fig. 3:**
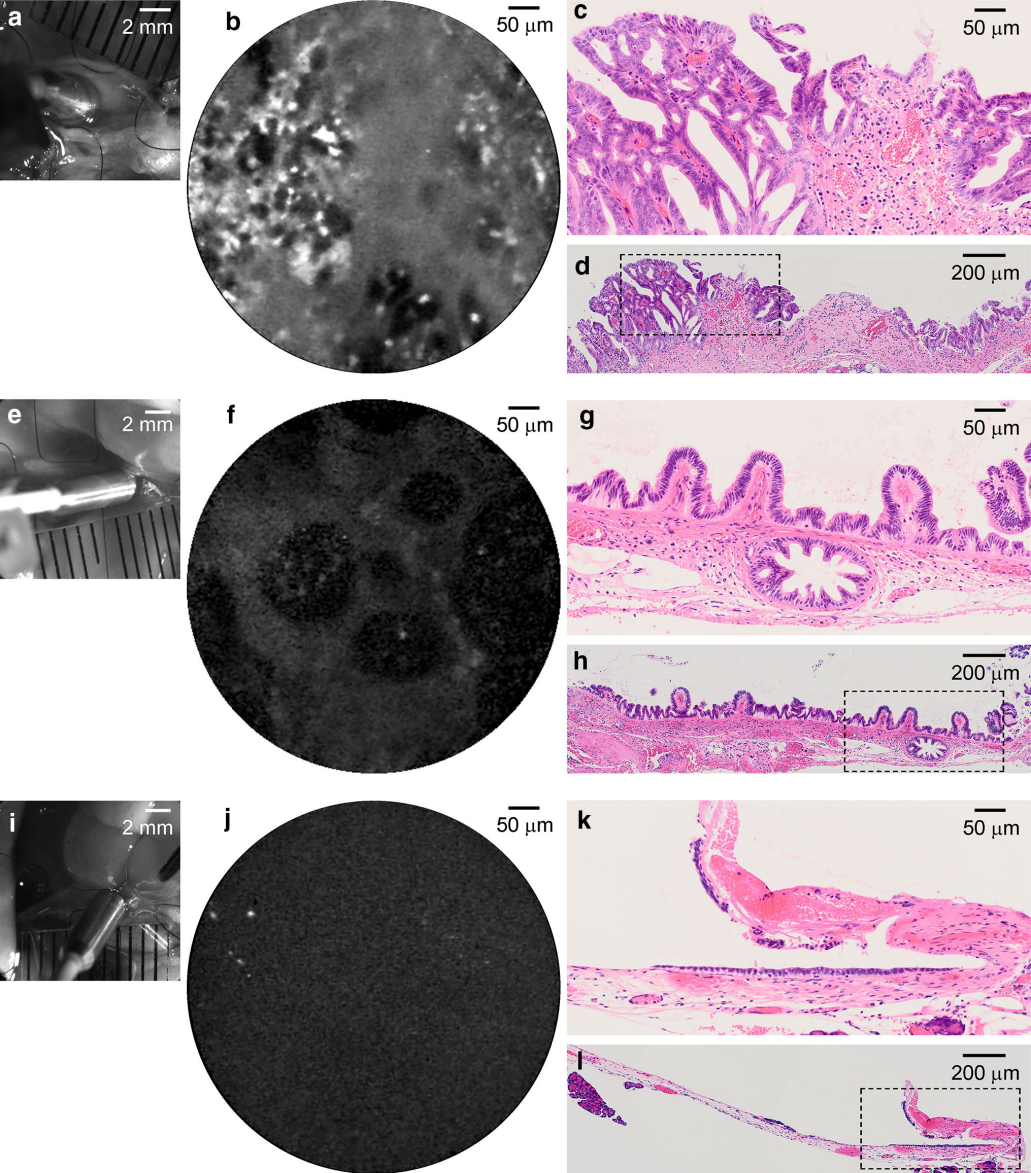
The fLG imaging of hamster bile duct by pCLE and the corresponding histological specimens. **a** A macro zoom microscopic image taken during fLG imaging of the biliary surface of a hamster in CDDB/BOP group. **b** A representative fLG image taken with pCLE at the site shown in **a** after topical administration of fLG into the bile duct. **c** A H&E section processed from the area corresponding to the site imaged in **a**, showing carcinoma in situ exhibiting highly disorganized papillary cytoarchitecture. **d** Similar to **c**, but in a reduced view. *Demarcated area* was magnified in (**c**). A more reduced view is shown in Online Resource 2. **e–h** Similar to (**a–d**), but of another animal in CDDB/BOP group. Reactive hyperplasia with round-shaped papillary protrusions (**g**) corresponded to dark clumps in **f**. For more details, see Online Resource 3. **I–l** Similar to **e–h**, but of an animal in normal control group. Simple epithelium is seen

The pCLE probe was then attached tightly and perpendicularly to the luminal surface of the bile duct (Fig. 2c, d). The epithelium of the common duct was carefully inspected from the liver side to the duodenum side with the assistance of a micromanipulator having multiple degrees of freedom. The average period required for completing survey of whole mid duct approachable with the pCLE probe was 21.1 ± 8.9 min (*n* = 24). The probe position in the bile duct was continuously monitored by a CCD camera mounted on the macro zoom microscope. Comparison of the CCD images of the probe end and individual time-matched images by pCLE was helpful to determine the region of interest (ROI) in histological sections (Fig. 3, see also Online Resources 2, 3).

The fLG imaging and following histopathological identification of the ROI were successfully conducted in totally 14 animals subjected to CDDB/BOP procedure in the experimental group. Of these, extrahepatic cholangiocarcinoma was developed in three animals in mid common duct (Fig. 3c, d, Online Resources 2, 4). In these animals, the biliary surface imaged by pCLE showed a fluorescence pattern consisting of characteristic bright spots and rounded or irregularly shaped dark clumps of various sizes (Fig. 3b, Online Resources 2, 4). According to the position of the pCLE probe monitored by a macro zoom microscope, histopathological sections corresponding to the area imaged by pCLE were determined, demonstrating carcinoma in situ with highly disorganized papillary cytoarchitecture in the area where the characteristic fLG fluorescence pattern was detected (Fig. 3a–d, Online Resource 2). A similar fluorescence pattern was detected in the specimen showing invasive carcinoma as well (Online Resource 4).

In two animals in the CDDB/BOP group, only hyperplastic atypia was detected throughout the extrahepatic bile duct. In these animals, relatively uniform fluorescence pattern was detected by pCLE in all biliary epithelia inspected. However, all remaining 12 animals in CDDB/BOP group showed more or less clear heterogeneity in the fLG fluorescence in the epithelia. The extent of heterogeneity in the fLG fluorescence pattern seemed to be correlated with histopathological grade in CDDB/BOP group (Fisher’s exact probability = 0.015 and 0.002 by two independent raters, respectively. Online Resource 5), although larger scale analysis should be required.

In one of the 12 animals, cholangiocarcinoma was detected only at the distal end of the bile duct (duodenum side), the pCLE probe therefore could not approach the lesion. In this animal, the epithelia in mid common duct exhibited relatively dark, large clumps in the pCLE image (Fig. 3f, Online Resource 3). These clumps corresponded well with rounded papillary protrusions in the epithelia composed of cells with well-maintained cell polarity (Fig. 3e–h, Online Resource 3). Contrasted to the CDDB/BOP group, all animals in the control group (*n* = 10) exhibited uniform fluorescence pattern (Fig. 3i, j), where relatively simple epithelia was seen in histology (Fig. 3 k, l), although large and dark single ellipse surrounded by homogeneous fluorescence [31] was occasionally seen in fLG imaging (data not shown).

**Fig. 4:**
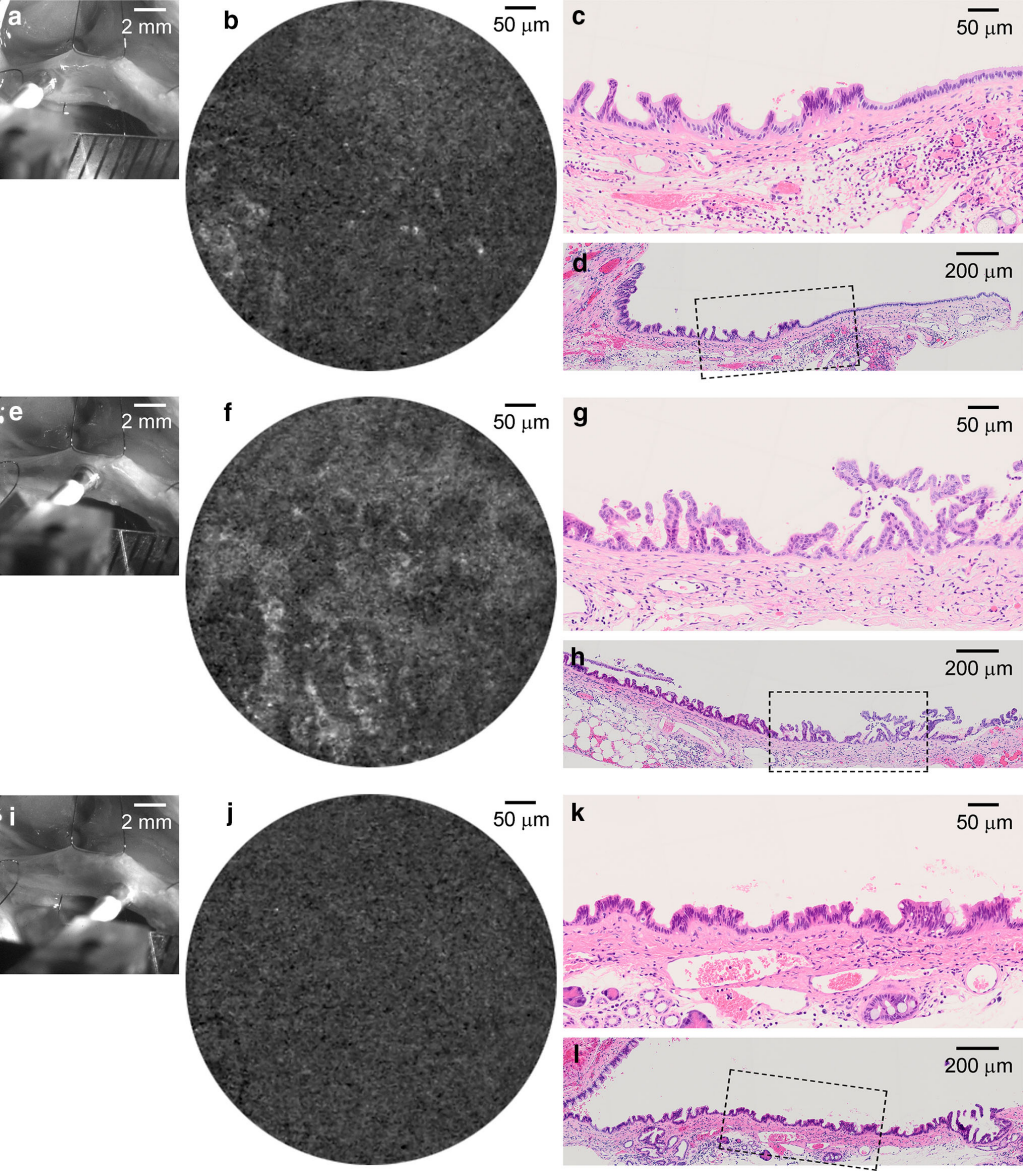
Autofluorescence images of the bile duct surface of a hamster in CDDB/BOP group taken in a condition with no fLG infusion, and the corresponding histological specimens. **a** A macro zoom microscopic image taken during autofluorescence imaging of the biliary surface. **b** A representative autofluorescence image taken with pCLE at the site shown in **a**. **c** H&E section processed from the area corresponding to the site imaged in **a**. The cytoarchitecture changes gradually from the *right to the left side*, non-neoplastic simple epithelium, hyperplasia with angular-shaped small papillary protrusion, to hyperplasia composed of thinner papillary structures. **d** A reduced view of **c**. Demarcated area was magnified. **e–h** Similar to **a–d**, but taken at a different site in the bile duct of the same animal. A heterogeneous but coarse fluorescence pattern was detected (**f**). Classified as dysplasia characterized by thin papillary protrusions composed of cells bearing heterogeneously enlarged nuclei with coarse granular chromatin (**g**). **i–l** Similar to **e–h**, but taken at a site much closer to duodenum side. Only dim homogeneous fluorescence is seen (**j**) for lesions diagnosed with hyperplasia composed of uniform angular-shaped papillary structures (**k**, **l**)

To assess an involvement of autofluorescence in fLG imaging, the bile duct of three animals subjected to CDDB/BOP procedure was imaged in a condition with no fLG infusion in the preliminary experiment (Fig. 4). As depicted, ambiguous (Fig. 4a–d) or almost no (Fig. 4i–l) fluorescence was detected in the epithelia corresponding to hyperplastic lesions. Although somewhat irregular autofluorescence pattern was detected in dysplastic lesions of the same animal, the characteristic fluorescence pattern typically seen in biliary epithelia after the fLG infusion has not been detected (Fig. 4e–h).

## Discussion

In the present study, we introduced a fluorescence imaging of a hamster model of extrahepatic cholangiocarcinoma by using fLG mixture composed of 2-NBDLG and 2-TRLG as the contrast agent for pCLE. Heterogeneous fluorescence patterns consisting of characteristic bright spots and dark clumps were detected from the biliary surface that was diagnosed with carcinoma in situ and with invasive adenocarcinoma, whereas no such fluorescence pattern was detected in control animals. Our findings may provide a basis to develop a method for real time imaging of cancerous/precancerous anomalies in extrahepatic bile duct by using the fLG mixture.

### Histopathology of hamster bile duct cancer model

In a pioneering study by Tajima and colleagues [29, 30], extrahepatic cholangiocarcinoma in mid common duct developed in 5/22 (22.7 %) of hamsters tested at 20 weeks after the beginning of BOP administration. Similar results were obtained in the present study; the extrahepatic cholangiocarcinoma developed in 3/14 (21.4 %) and 2/13 (15.4 %) of hamsters for the experimental and preliminary group, respectively, tested at 17–29 weeks after the beginning of BOP administration. Although making cholangiocarcinoma in the mid common duct approachable by pCLE probe took over several months, this hamster model might provide valuable information on understanding of cholangiocarcinoma [32]. For example, cytoarchitectonical changes along with the biliary surface detected in the present study from simple epithelia, hyperplastic to dysplastic papillary protrusion, carcinoma in situ, to invasive carcinoma (Fig. 1b–d) might be consistent to a recent proposal wherein the intraductal papillary neoplasm represents a precursor region of invasive carcinoma [33].

### Correlation between fLG fluorescence and histopathology in the hamster bile duct

The large and dark clumps (or island pattern) in vague background detected in fLG images most probably reflect round-shaped papillary protrusions, composed of cells with well-maintained cell polarity (Fig. 3f–h, Online Resource 3) [31]. In contrast, the fluorescence pattern consisting of small bright spots associated with irregular dark clumps with various sizes appeared to correspond to highly disorganized epithelia including cells showing loss of polarity and coarse granular chromatin (Fig. 3b–d, Online Resource 2).

Dark clump-like structures in the pCLE image obtained in CDDB/BOP group resembled those reported in bile duct of human patients where fluorescein was used as a contrast agent for pCLE [3–7, 10]. However, careful investigation should be required for the comparison, since 2-NBDLG is taken up into tumor cells, whereas fluorescein is not taken up into cells [3–7, 10, 27].

Contrasted to CDDB/BOP group, only uniform fluorescence pattern was obtained in fLG imaging in normal control group, implying that fLG is a unique candidate tracer reflecting cellular architecture. The present proposal for identifying cholangiocarcinoma by pCLE in vivo somewhat resembles the fluorescence intensity criteria using 2-NBDG for detecting Barrett’s dysplasia ex vivo [34]. It could well be speculated, however, use of 2-NBDLG provides more specific information on neoplastic anomalies while minimizing background uptake compared to the case when using 2-NBDG [27]. Further study should be conducted for obtaining more precise correspondence between the fluorescence images and histological architectures. Indeed, we could not identify the detection area of the pCLE probes used (0.6 mm in diameter, one-fourth of the outer diameter of Demo probe and one-third of Alveoflex probe) precisely from the information obtained by the macro zoom microscope. To correlate the ROI in the fLG fluorescence image with later histopathology at the single cell level, development of a special pCLE probe and/or some positioning devices would be needed.

### Identification of 2-NBDLG and 2-TRLG fluorescence

Since the clinical pCLE apparatus used in the present study can detect fluorescence only in a single channel, 2-NBDLG and 2-TRLG fluorescence could not be separately visualized by the endoscope. Although we have tried to evaluate 2-NBDLG and 2-TRLG fluorescence by the macro zoom microscope, low magnification macro lens used in the present study failed to distinguish individual cellular states in the mucosa due to its limited horizontal and depth resolution. As such, it is not excluded that fluorescence signals detected might include not only 2-NBDLG uptake into tumor cells but also 2-TRLG permeation to damaged and/or inflammatory cells.

Another important issue to be discussed is how to discriminate 2-NBDLG fluorescence and autofluorescence originated from such as elastin, collagen, and flavoprotein [35, 36]. Indeed, it has been reported that bile duct ligation causes many proliferating myofibroblasts secreting high amount of elastin in the vicinity of the epithelial layer [37]. Although autofluorescence appears to provide relatively coarse spatial information distinct from those detected in fLG imaging (Fig. 4), one of more essential strategies for discriminating the fLG fluorescence from autofluorescence would be to take images before and after application of the fLG mixture in two separate wavelength ranges optimized for 2-NBDLG and 2-TRLG fluorescence [27]. Development of such system is currently underway in our laboratory. It is generally a difficult task to separately identify the fluorescence signal derived from cancerous/precancerous lesions and that originated from inflammation [10, 38]. Imaging of inflammatory mucosa with no infiltration of carcinoma will be required as a control using a dual wavelengths endoscope system with fine spatial resolution.

The molecular mechanism underlying the 2-NBDLG uptake into tumor cells has yet to be clarified. Although uptake of 2-NBDG into mammalian cells can occur through GLUTs [12, 13], an involvement of non-GLUT-mediated mechanism has been suggested for the uptake of 2-NBDLG into tumor cells [27]. 2-NBDLG uptake into cultured tumor cells occurred in three-dimensionally accumulating spheroids consisting of heterogeneous cells having nuclei of varying N/C ratio [27]. It may be therefore interesting to further investigate if 2-NBDLG is taken up into not only cancerous but also pre-cancerous cells in the bile duct.

A highly disorganized fluorescence pattern obtained from CDDB/BOP group was very different from that in normal group. Using the derivatives of GLUT-unrecognizable l-glucose as contrast agents for pCLE might well detect extrahepatic cholangiocarcinoma while minimizing the potential toxicity and the background uptake into normal cells. Bacterial reverse mutation tests and extended single oral dose toxicity studies for 2-NBDLG and 2-TRLG have been successfully conducted in our laboratory according to Good Laboratory Practice regulations, making expectations toward their use in humans to grow. Further studies are required to fully elucidate the potential of the fLG mixture for visualizing bile duct cancer in vivo.

## Electronic supplementary material

Below is the link to the electronic supplementary material.

Supplementary material 1 (PDF 978 kb)
