# Supplementary material for: Imaging hamster model of bile duct cancer in vivo using fluorescent l-glucose derivatives
Source: Hum Cell. 2016 Feb 3;29:111–21. doi: 10.1007/s13577-015-0131-5 (PMC4930486; doi:10.1007/s13577-015-0131-5)
Supplement: Supplementary file 1 — Supplementary material 1 (PDF 978 kb) [file 13577_2015_131_MOESM1_ESM.pdf]

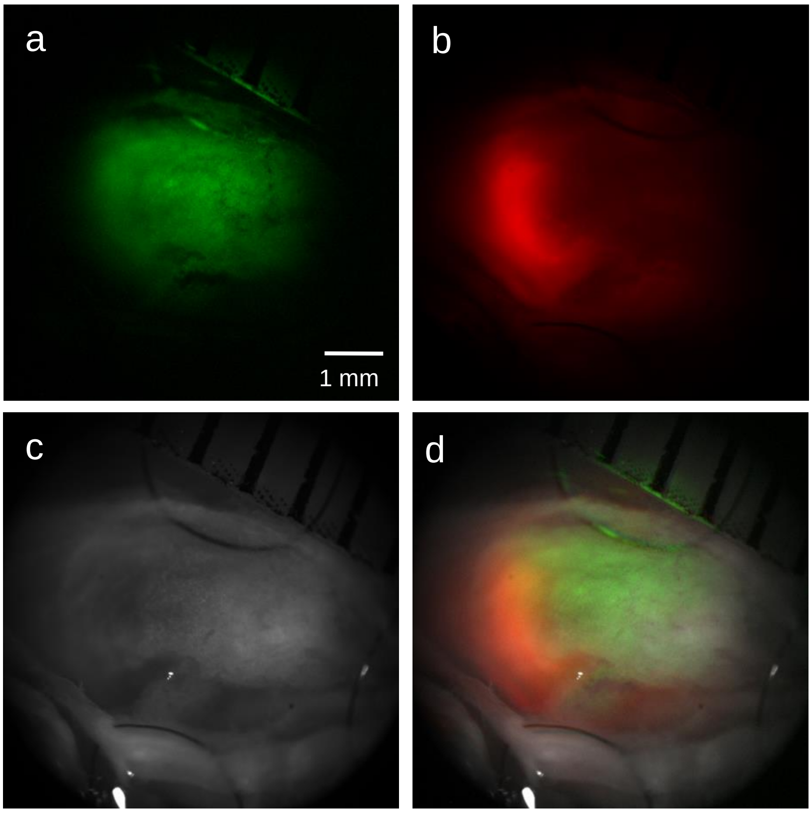

Online Resource 1. Macroscopic views of hamster bile duct depicted in **Fig.3a-d.** Fluorescent images taken by a macro zoom microscope expressed in the *green* (**a**, 545 nm) and the *red* (**b**, 641 nm) pseudcolor, reflecting 2-NBDLG and 2-TRLG, respectively. **c** Bright-field image. **d** Overlay of the *green* (**a**), *red* (**b**) and bright-filed (**c**) images. A macro lens (x4, NA 0.2) was used at x2 zoom ratio. 2-TRLG fluorescence suggested an occurrence of non-specific entry of the agent in the area. However, such area was not excluded from the present analysis, since precise identification of 2-TRLG-positive cells was difficult by low magnification, high depth of field lens used. Scale bar is common to all panels.

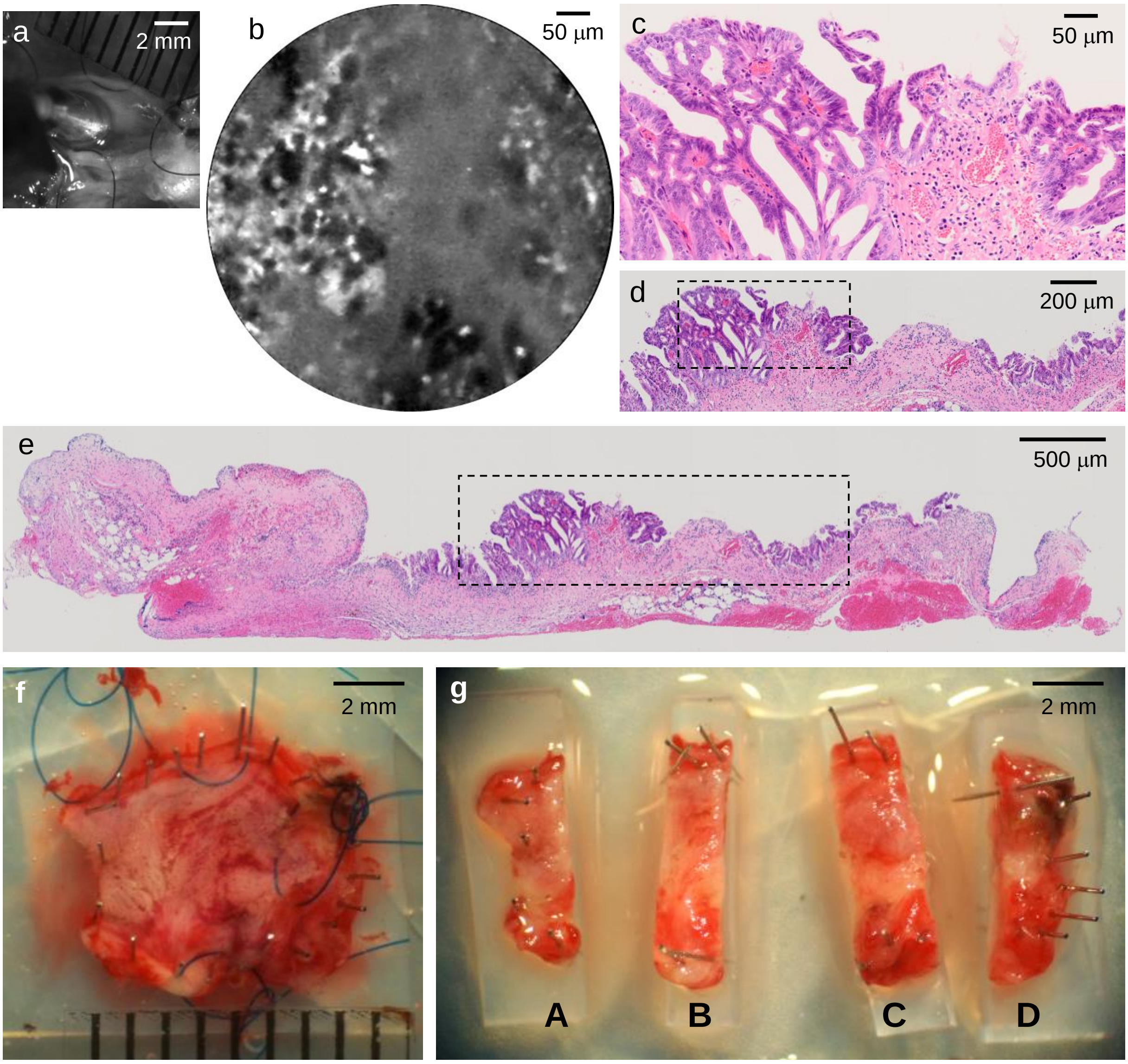

Online Resource 2. The fLG imaging by pCLE, the corresponding histological sections, and excised tissues of hamster bile duct depicted in **Fig. 3a-d**. **a** A macro zoom microscopic image of the bile duct taken during fLG imaging by pCLE. **b** A representative fLG image by pCLE taken at the site shown in (**a**) after topical administration of fLG into the bile duct. **c** H&E section processed from the area corresponding to the site imaged in (**a**), showing carcinoma *in situ* exhibiting highly disorganized papillary cytoarchitecture. **d** Similar to (**c**), but in a reduced view. Demarcated area was magnified in (**c**). **e** Similar to (**d**), but in a more reduced view. **f** Excised bile duct in Krebs-Ringer buffer. **g** Bile duct being cut into four pieces in formaldehyde for making histological sections shown above.

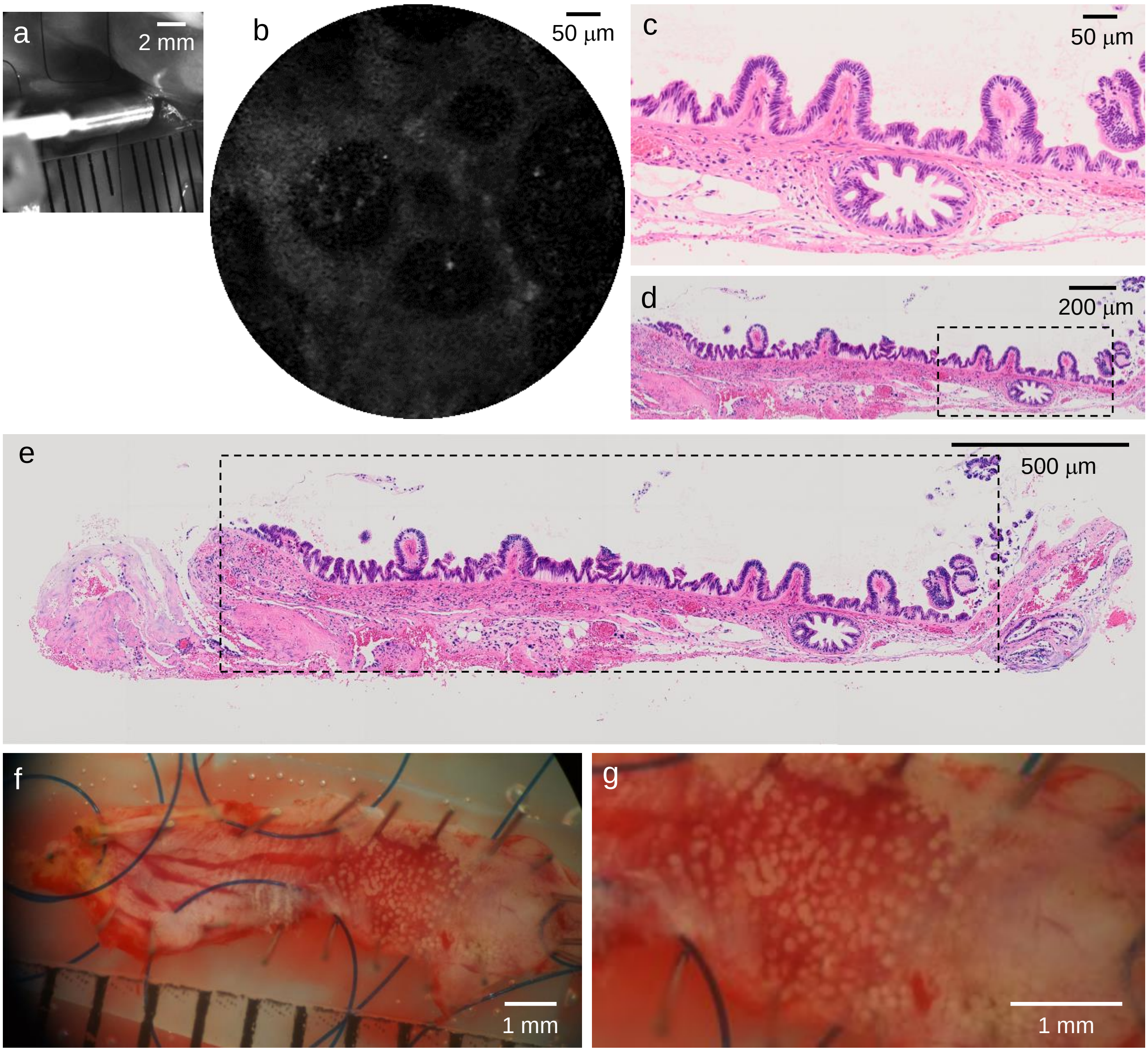

Online Resource 3. The fLG imaging by pCLE, the corresponding histological sections, and excised tissues of hamster bile duct depicted in **Fig. 3e-h**. **a** A macro zoom microscopic image of the bile duct taken during fLG imaging by pCLE. **b** A representative fLG image by pCLE taken at the site shown in (**a**) after topical administration of fLG into the bile duct. **c** H&E section processed from the area corresponding to the site imaged in (**a**), showing reactive hyperplasia with round-shaped papillary protrusions corresponded to dark clumps in (**b**). **d** Similar to (**c**), but in a reduced view. Demarcated area was magnified in (**c**). **e** Similar to (**d**), but in a more reduced view. Angular-shaped small papillary structures consisting of mildly dysplastic cells are seen as well. **f** Excised bile duct in Krebs-Ringer buffer. **g** Similar to (**f**), but in a more magnified view.

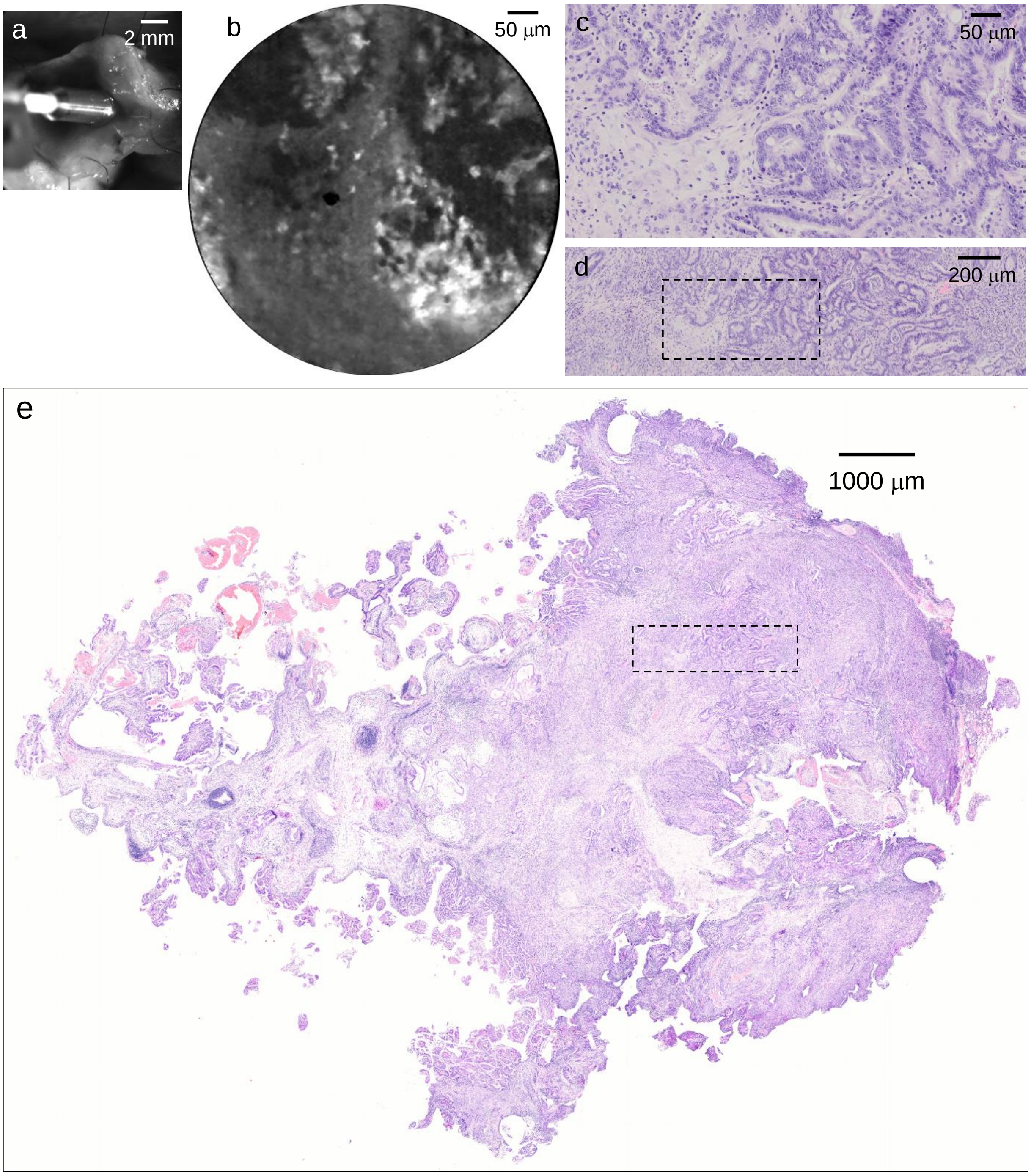

Online Resource 4. The fLG imaging by pCLE and the corresponding horizontal sections of the bile duct of a hamster in CDDB/BOP group. **a** A macro zoom microscopic image of the bile duct taken during fLG imaging by pCLE. **b** A representative fLG image by pCLE taken at the site shown in (**a**) after topical administration of fLG into the bile duct. Similar to **Online Resource 2b**, fluorescence pattern consisting of bright spots and irregular dark clumps of various sizes is seen. **c** H&E section processed from the area corresponding to the site imaged in (**a**), showing invasive adenocarcinoma with highly disorganized cytoarchitecture. **d** Similar to (**c**), but in a reduced view. Demarcated area was magnified in (**c**). **e** Similar to (**d**), but in a more reduced view.

Rater 1

Relationship between histopathological diagnosis and heterogeneity in the fLG fluorescence

	Heterogeneity in the fLG fluorescence		
Diagnosis	Marked	Moderate	Mild/Homogeneous
Carcinoma	3	0	0
Dysplasia	5	4	0
Hyperplasia	0	0	2
Summation	8	4	2

Fisher’s exact probability = 0.015 (< 0.05)

Rater 2

Relationship between histopathological diagnosis and heterogeneity in the fLG fluorescence

	Heterogeneity in the fLG fluorescence		
Diagnosis	Marked	Moderate	Mild/Homogeneous
Carcinoma	3	0	0
Dysplasia	3	6	0
Hyperplasia	0	0	2
Summation	6	6	2

Fisher’s exact probability = 0.002 (< 0.01)

Online Resource 5. The Freeman-Hamilton extension of Fisher’s exact test was conducted for analyzing the relationship between heterogeneity in fLG fluorescence pattern and histopathological diagnosis of fLG-administered animals in CDDB/BOP group (n = 14). For this purpose, the diagnosis was categorized into three groups; carcinoma including invasive one and carcinoma *in situ* (carcinoma), dysplasia, and hyperplasia including reactive one and mild dysplasia (hyperplasia). The heterogeneity in the fLG fluorescence was classified into three groups; marked, moderate, and mild (or homogeneous). Two raters independently classified the same series of fLG images into the three levels of heterogeneity.
